# Degradation of lignin β‐aryl ether units in *Arabidopsis thaliana* expressing *LigD*,* LigF* and *LigG* from *Sphingomonas paucimobilis* SYK‐6

**DOI:** 10.1111/pbi.12655

**Published:** 2016-11-29

**Authors:** Ewelina Mnich, Ruben Vanholme, Paula Oyarce, Sarah Liu, Fachuang Lu, Geert Goeminne, Bodil Jørgensen, Mohammed S. Motawie, Wout Boerjan, John Ralph, Peter Ulvskov, Birger L. Møller, Nanna Bjarnholt, Jesper Harholt

**Affiliations:** ^1^ Plant Biochemistry LaboratoryDepartment of Plant Biology and Environmental SciencesUniversity of CopenhagenFrederiksberg CDenmark; ^2^ Department of Plant Biotechnology and BioinformaticsGhent UniversityGhentBelgium; ^3^ Department of Plant Systems BiologyVIBGhentBelgium; ^4^ Department of Biochemistry and DOE Great Lakes Bioenergy Research CenterWisconsin Energy InstituteMadisonWIUSA; ^5^ Section for Plant GlycobiologyDepartment of Plant Biology and Environmental SciencesUniversity of CopenhagenFrederiksberg CDenmark; ^6^ Carlsberg Research LaboratoryCopenhagenDenmark

**Keywords:** biofuel, lignin modification, bacteria, *Sphingomonas paucimobilis*, Ligβ‐aryl ether, saccharification yield

## Abstract

Lignin is a major polymer in the secondary plant cell wall and composed of hydrophobic interlinked hydroxyphenylpropanoid units. The presence of lignin hampers conversion of plant biomass into biofuels; plants with modified lignin are therefore being investigated for increased digestibility. The bacterium *Sphingomonas paucimobilis* produces lignin‐degrading enzymes including LigD, LigF and LigG involved in cleaving the most abundant lignin interunit linkage, the β‐aryl ether bond. In this study, we expressed the *LigD*,* LigF* and *LigG* (*LigDFG*) genes in *Arabidopsis thaliana* to introduce postlignification modifications into the lignin structure. The three enzymes were targeted to the secretory pathway. Phenolic metabolite profiling and 2D HSQC NMR of the transgenic lines showed an increase in oxidized guaiacyl and syringyl units without concomitant increase in oxidized β‐aryl ether units, showing lignin bond cleavage. Saccharification yield increased significantly in transgenic lines expressing *LigDFG*, showing the applicability of our approach. Additional new information on substrate specificity of the LigDFG enzymes is also provided.

## Introduction

Lignin is one of the most abundant biopolymers in the world. Together with cellulose, hemicelluloses, pectin and additional minor components that constitute the plant cell wall, lignin offers mechanical strength and affords protection against pathogens. The hydrophobic properties of lignin make it a crucial polymer controlling water conduction (Bonawitz and Chapple, 2010). During the polymerization process, lignin fills up spaces between polysaccharides in the secondary plant cell wall and may be covalently cross‐linked to some of these. The properties of lignin impede enzymatic lignocellulose deconstruction and thus constitute a major bottleneck in biofuel production (Pauly and Keegstra, 2010; Van Acker *et al*., 2013; Vanholme *et al*., 2013a). Energy‐demanding and expensive pretreatment is required to facilitate the access of hydrolytic enzymes to the polysaccharides in the cell wall. Modification of the cell wall structure in ways that ease deconstruction without compromising the agronomic performance of the crop plant is therefore an important research topic. However, plants with large reductions in lignin content exhibit reduced growth, fitness and development. Altering lignin composition and structure might therefore be a better strategy to improve the efficiency of biomass processing (Bonawitz and Chapple, 2010; Li *et al*., 2008; Ralph, 2007; Sederoff *et al*., 1999; Vanholme *et al*., 2008, 2012).

Lignin is a complex aromatic polymer derived mainly from three monolignols: *p*‐coumaryl alcohol, coniferyl alcohol and sinapyl alcohol. When incorporated into lignin, these monolignols give rise to the *p‐*hydroxyphenyl (H), guaiacyl (G) and syringyl (S) units, respectively. Once monolignols are synthesized in the cytoplasm, they are exported to the apoplast, activated to form radicals by the action of peroxidases or laccases and thereby subjected to radical coupling polymerization reactions. The most abundant interunit linkage type, formed primarily by the coupling of a monolignol (at its β‐position) to the phenolic end (at its 4–O‐position) of a growing polymer chain, is β–O–4 referring to an ether linkage between the aliphatic side‐chain of one monolignol and the phenolic moiety of another. Additional linkage types formed are β–5, 5–5, β–β and 4–O–5 (Boerjan *et al*., 2003; Ralph *et al*., 2004).

**Figure 1:**
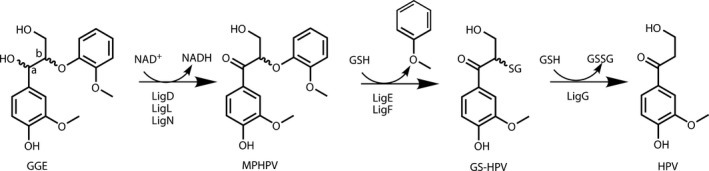
Pathway for degradation of β–O–4‐linked units by Lig enzymes from *S. paucimobilis* exemplified with the model compound GGE (Masai *et al*., 2003). GGE, guaiacylglycerol‐β‐guaiacyl ether; MPHPV, α‐(2‐methoxyphenoxy)‐β‐hydroxypropiovanillone; GS‐HPV, α‐glutathionyl‐β‐hydroxypropiovanillone; HPV, β‐hydroxypropiovanillone; GSH, glutathione.

The ability of microorganisms to degrade lignin has been extensively studied. White‐rot and brown‐rot fungi are involved in high‐molecular‐mass lignin degradation whereas bacteria like *Sphingomonas paucimobilis* SYK‐6 degrade low‐molecular‐mass lignin fragments and small oligomers (Kirk and Farrell, 1987; Masai *et al*., 2007). The metabolism of *S. paucimobilis* has been evolutionarily adapted to allow the bacterium to grow on lignin‐derived compounds. This involves development of enzymatic machinery to fully degrade different types of units. Reductive cleavage of β‐aryl ether units has been demonstrated *in vitro* using guaiacylglycerol‐β‐guaiacyl ether (GGE) as a model substrate (Masai *et al*., 2003) (Figure 1). The β–O–4‐linked moieties are reductively cleaved in the course of a three‐step sequence that involves initial dehydrogenation (catalysed by LigD, LigL and LigN enzymes), followed by reductive cleavage of the ether linkage catalysed by a glutathione S‐transferase (LigE or LigF), involving the formation of a glutathione conjugate, and finally reductive cleavage of this conjugate, producing the monomer and oxidized glutathione in a step using additional glutathione, catalysed by a glutathione lyase (LigG). The enzymes catalysing these processes are highly stereospecific. The GGE substrate exists as four enantiomers. The α*R*‐GGE enantiomers are dehydrogenated by LigD, whereas the αS‐GGE enantiomers are dehydrogenated by LigL or LigN. The LigF enzyme conjugates the β*S* enantiomer of α‐(2‐methoxyphenoxy)‐β‐hydroxypropio‐vanillone (MPHPV) with glutathione (GSH) whereas the β*R* enantiomer is conjugated by LigE (Sato *et al*., 2009). The substrate specificity assays were mainly performed on guaiacyl dimers (Gall *et al*., 2014a; Masai *et al*., 2007), but *in vitro* the Lig enzymes are also capable of degrading synthetic high‐molecular‐mass polymers mimicking the structure of lignin (Sonoki *et al*., 2002).

A recent paper described an attempt to engineer *LigD* into the plant with the idea of creating benzylic‐oxidized lignins that might chemically degrade more readily (Tsuji *et al*., 2015). In this study, the codon‐optimized *S. paucimobilis* SYK‐6 genes encoding the complete set of lignin‐degrading enzymes LigD, LigF and LigG (henceforth abbreviated LigDFG) were inserted into the Arabidopsis genome by stable transformation using transit peptides targeting each of the heterologous proteins to the secretory pathway.

## Results

### Transient expression of *LigDFG* in tobacco

The *S. paucimobilis LigD*,* LigF*,* LigG* gene sequences were codon‐optimized for expression in Arabidopsis. The bacterial *Lig* genes have a high GC content (approx. 60%), and as a result of codon optimization, the GC content was lowered from 64% to 54% in *LigD*, from 61% to 51% in *LigF* and from 63% to 54% in *LigG*. With the goal of modifying the cell wall, proteins involved in the LigDFG pathway had to be targeted to the secretory pathway to achieve localization in the apoplast. To achieve this, each separate protein was fused with the barley α‐amylase signal peptide (amy) (Rogers, 1985) or potato proteinase inhibitor II (ppi) (Liu *et al*., 2004). These transit peptides have previously been used to target heterologous proteins to the secretory pathway (Borkhardt *et al*., 2010; Buanafina *et al*., 2010; Herbers *et al*., 1995). For expression of the entire pathway, *LigD*,* LigF* and *LigG* were expressed in tri‐cistronic constructs in which the three coding sequences were linked by the *2A* gene sequence coding for a self‐processing polypeptide similar to the one found in the *Foot‐and‐mouth disease virus* (El Amrani *et al*., 2004). Successful expression of multiple proteins in plants from constructs and targeting them to different cellular compartments has previously been reported, including apoplastically localized cell wall degrading enzymes (El Amrani *et al*., 2004; de Felipe, 2002; Øbro *et al*., 2009). To test whether the tri‐cistronic constructs *amy‐LigD‐2A‐amy‐LigF‐2A‐amy‐LigG* (*amyDFG*) and *ppi‐LigD‐2A‐ppi‐LigF‐2A‐ppi‐LigG* (*ppiDFG*) yielded active proteins *in planta*, they were transiently expressed in *Nicotiana benthamiana* (tobacco) by infiltration into the leaves. Enzymatic assays with proteins extracted from the infiltrated leaves using GGE or MPHPV as substrate showed formation of hydroxypropiovanillone (HPV) from both (Figure S1). HPV is the expected product formed from GGE by the action of LigDFG (Figure 1). This demonstrated that the heterologously expressed proteins were functional. The intermediate MPHPV was also formed from GGE in protein extracts from both constructs, whereas α‐glutathionyl‐β‐hydroxypropiovanillone (GS‐HPV) was only detected in the enzymatic assay with proteins from plants expressing *amyDFG* (Figure S1). Presumably, the steady‐state pool size of GS‐HPV in plants expressing *ppiDFG* was below the detection limit determined by the experimental design.

### Stable *Arabidopsis* transformants expressing either LigD, LigF or LigG

In order to investigate the *in vivo* catalytic properties of the individual enzymes in Arabidopsis and to optimize the protein extraction method, Arabidopsis plants were transformed with constructs to separately express either *LigD, LigF* or *LigG*, each with the *amy* or *ppi* signal peptide code, under the control of the 35S promotor.

GGE, MPHPV and GS‐HPV were used as a substrate to monitor the catalytic function of LigD, LigF and LigG, respectively, using crude protein extracted from leaves of the transformed plants (see pathway in Figure 1). Leaves were used as the enzyme source due to the ease of sufficient amounts of protein by extraction and their formation in the early part of the growth cycle thereby reducing the growth period. All genes were expressed under the control of the 35S promotor which would be expected to result in comparable protein accumulation in other tissues. Because GS‐HPV was not available as a chemical standard, this reference compound was produced enzymatically by incubation of MPHPV with protein extracts of *Escherichia coli* expressing LigF. The reaction mixture obtained contained mainly GS‐HPV and was used to test for LigG activity. The enzyme assays with the majority of the transformed lines indeed produced the expected products (Figure S2). The total number of lines identified that were positive for insertion of the transgene as detected by PCR and for the expected corresponding enzyme activity were three lines expressing *amy‐LigD* (*amyD*), two lines expressing *amy*‐*LigF* (*amyF*), two lines expressing *amy‐LigG* (*amyG*), two lines expressing *ppi‐LigD* (*ppiD*) and two lines expressing *ppi‐LigF* (*ppiF*).

Interestingly, HPV was also detected in plants expressing only *LigF* when assayed using MPHPV as substrate (Figure S2). In the control samples where MPHPV was incubated with protein extracted from wild‐type plants, no formation of HPV was detected. This indicated that an endogenous LigG‐like activity was present in wild‐type Arabidopsis plants or that LigF possessed LigF as well as LigG activity. No HPV could be detected when wild‐type protein extracts were incubated with GS‐HPV, supporting the contention that LigF possesses additional LigG activity, but this needs to be studied in more detail.

The results showed that it was possible to monitor the activity of the individual enzymes when expressed with either the amy or ppi signal peptides and that the optimized protein extraction and assay conditions were adequate as a screening method for identification of plants successfully expressing the entire LigDFG pathway after transformation of Arabidopsis with the tri‐cistronic constructs.

### Engineering the entire LigDFG pathway into Arabidopsis

**Figure 2:**
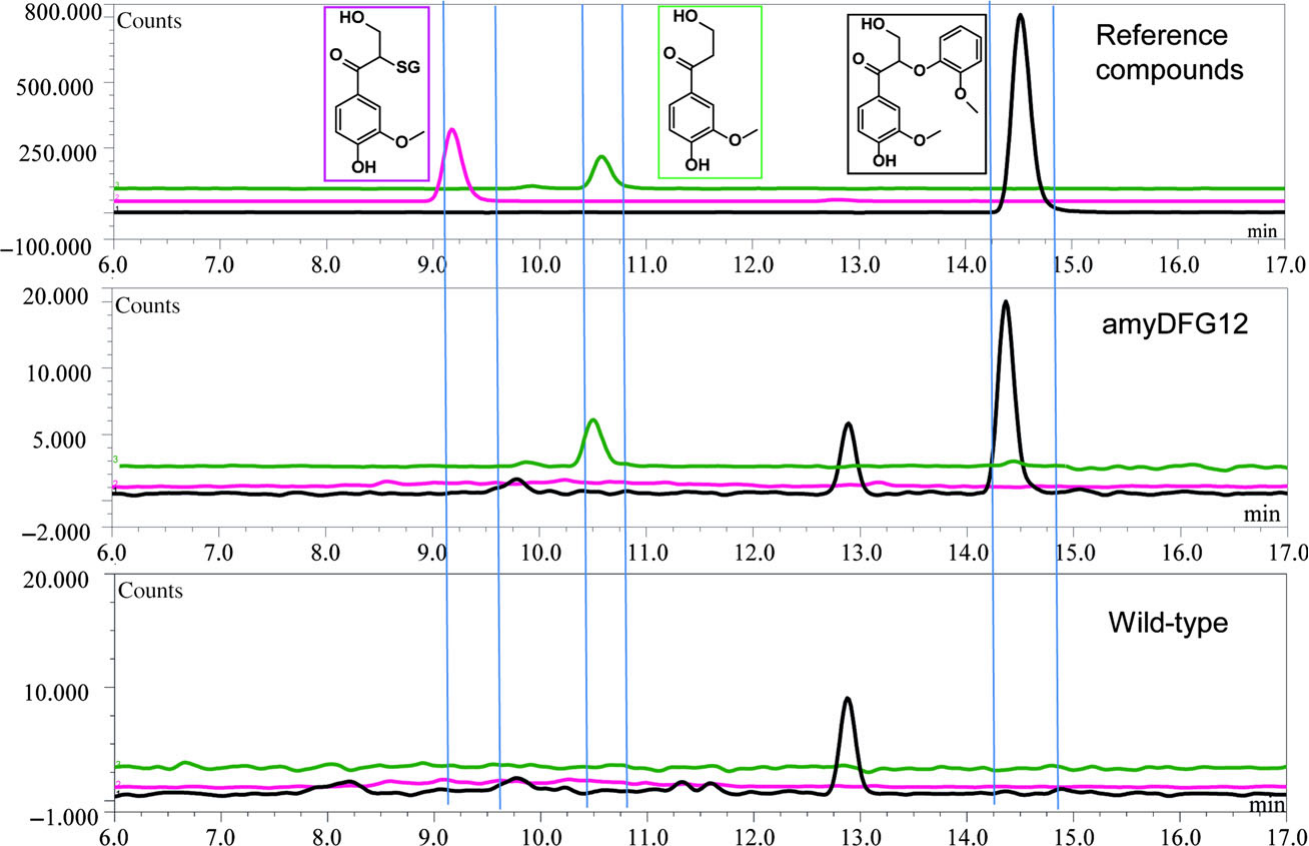
Detection of LigDFG catalysed products and intermediates formed in transgenic Arabidopsis plants expressing *LigDFG*. The chromatogram in the upper panel shows the retention times of the reference compounds. In the panels below, the metabolism of guaiacylglycerol‐β‐guaiacyl ether following incubation with leaf protein extracts from a transgenic line and wild‐type plants is shown. Black line: Extracted ion chromatogram (EIC) for *m*/*z* 319 recorded to detect formation of α‐(2‐methoxyphenoxy)‐β‐hydroxypropio‐vanillone. Pink line: EIC for *m*/*z* 502 recorded to detect formation of GS‐HPV. Green line: EIC *m*/*z* 197 recorded to detect formation of HPV. LigDFG, enzymes LigD, LigF and LigG.

To metabolically engineer lignin *in planta* using the LigDFG pathway*, amyDFG* and *ppiDFG* constructs were stably transformed into Arabidopsis. Initially, positive first‐generation transformants obtained using the tri‐cistronic constructs were identified by PCR. The positive transformants were then screened using the optimized assays for enzymatic activity using protein extracts from leaves. LigD activity was detected in seven and five independent transformants expressing *amyDFG* and *ppiDFG,* respectively, as demonstrated by the observed enzymatic conversion of GGE to MPHPV. Three of the transformants exhibiting LigD activity (*amyDFG10*,* amyDFG12* and *ppiDFG1*) also showed LigFG activity as demonstrated by formation of HPV (Figure 2). These independent transformed lines were selected for further characterization.

**Figure 3:**
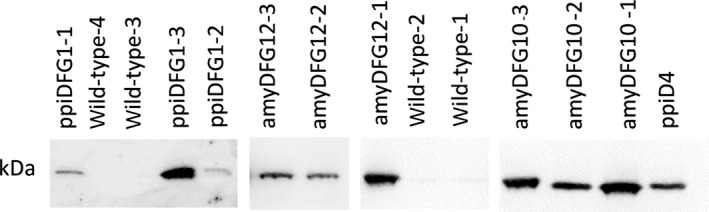
Detection of LigD protein in *LigDFG* transgenic lines (third‐generation T3) as monitored by Western blotting with anti‐LigD antibody. No bands corresponding to unsuccessful 2A proteolytic cleavage, between LigD and LigF could be detected, as only the shown 31.9 kDa band could be detected with anti‐LigD antibody. The figure is a composite of several blots.

Polyclonal antibodies against LigD were raised in rabbits and used to investigate the abundance of LigD protein in the transgenic *amyDFG10*,* amyDFG12* and *ppiDFG1* plants. By use of the polyclonal antibodies, LigD was detected in crude protein extracts from rosette leaves of *amyDFG10*,* amyDFG12* and *ppiDFG1* plants and in *ppiD4* (*LigD*‐expressing) plants that were used as a positive control (Figure 3). No bands corresponding to unsuccessful 2A proteolytic cleavage, between LigD and LigF could be detected, as only a single band of 31.9 kDa could be detected with anti‐LigD antibody. No LigD was detected in the protein extract from wild‐type rosette leaves used as a negative control (Figure 3). The three independent biological replicates of *amyDFG10* showed a high and consistent abundance of LigD, whereas substantial variation in LigD abundance was observed among the three biological replicates of *ppiDFG1* and among the three biological replicates of *amyDFG1*2 (Figure 3).

### Lignin characterization by 2D NMR

**Figure 4:**
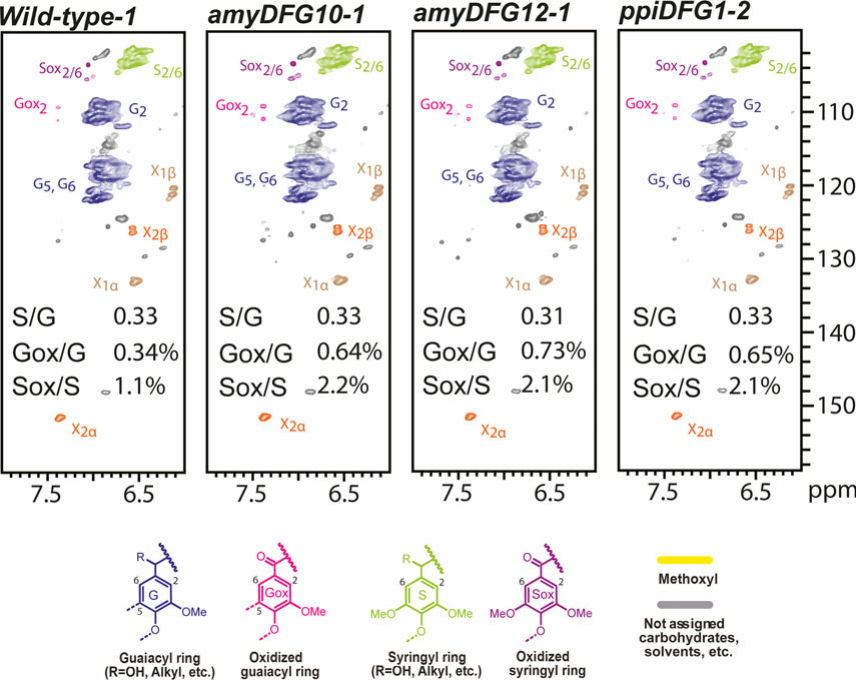
Aromatic region of partial 2D HSQC NMR spectra obtained by analysis of three biological replicates of the *LigDFG* lines and wild type. A single representative spectrum from each line is shown. The content of particular units is the average of the three replicates. See Table S3 for details.

**Figure 5:**
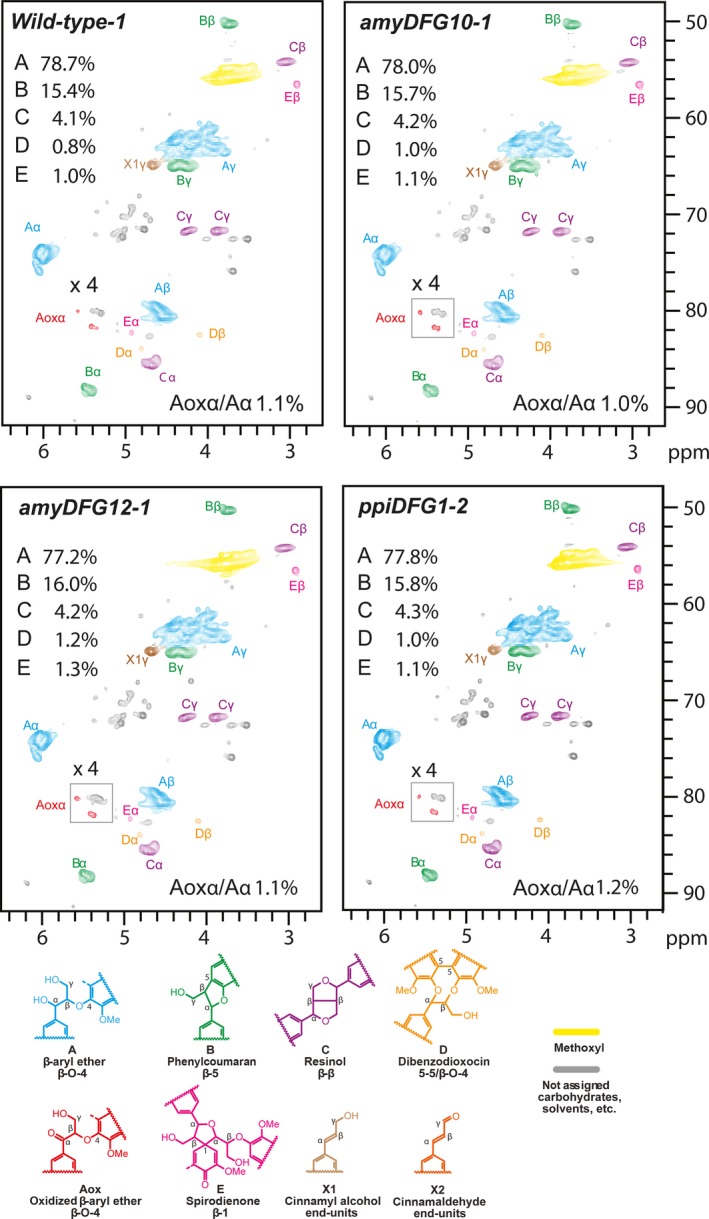
Aliphatic region of partial 2D HSQC NMR spectra obtained by analysis of three biological replicates of the *LigDFG* lines and wild type. A single representative spectrum from each line is shown. The content of particular units is the average of the three replicates. See Table S3 for details.

NMR is a powerful tool for qualitative analysis and for quantifying the relative amounts of the different linkage types and lignin building blocks present in lignin (Lu and Ralph, 2011; Mansfield *et al*., 2012; Morreel *et al*., 2010). The signals in the aliphatic region of 2D HSQC NMR spectra depict the linkage‐type distribution, whereas the signals in the aromatic region can be used to deduce the monomeric lignin composition. Enzymatic lignins extracted from dried, mature but still green inflorescence stems of T3 *amyDFG10*,* amyDFG12*,* ppiDFG1* and wild‐type plants were used for the analyses. The relative amounts of different lignin substructures between the wild‐type and transgenic lines were obtained from volume integration of the HSQC contours (Table S3), and the summary of the results is shown in Figures 4 and 5. In general, the analyses revealed that the overall monomeric and linkage‐type composition of the transgenic plants were similar to those in the wild type. However, the *LigDFG* lines showed a statistically significant twofold increase in α‐keto‐G (Gox) and α‐keto‐S (Sox) units. The Gox/G ratio was increased from 0.34% in wild type to 0.64%, 0.73% and 0.65% in the *amyDFG10*,* amyDFG12* and *ppiDFG1* lines, respectively (Figure 4). The Sox/S ratio was 1.09% in wild type and increased to 2.16%, 2.13% and 2.13% in the *amyDFG10*,* amyDFG12* and *ppiDFG1* lines, respectively. The observed higher abundance of Gox and Sox might be logically explained by LigD activity (Tsuji *et al*., 2015). However, the increased amounts of Gox and Sox units observed in the aromatic region were not reflected in the aliphatic region; there were no significant differences in oxidized β‐aryl ether units (Aox) as deduced from the Aox/A ratio, showing that the additional oxidized G and S units (Gox and Sox) were not bound in such units (Figures 4 and 5).

### Phenolic metabolite profiling

**Figure 6:**
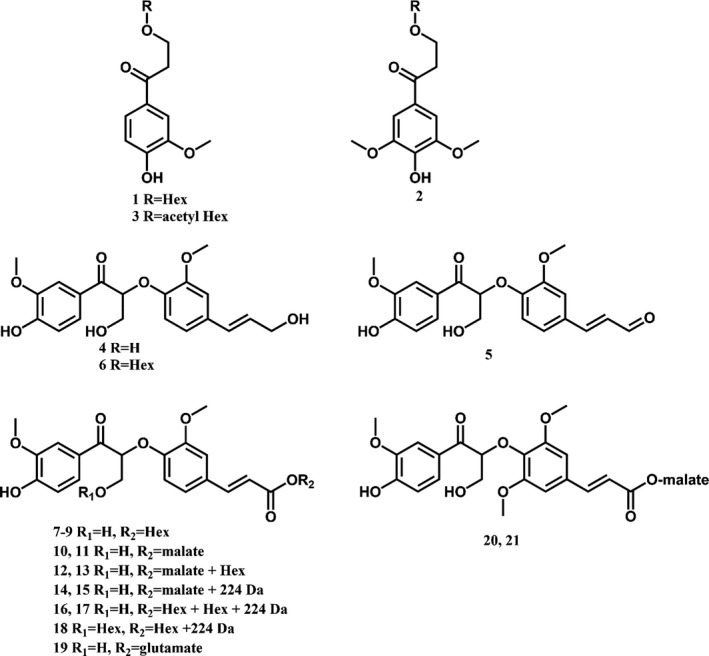
Structures of phenolic compounds which show significant differences between their abundance in *LigDFG* transgenic Arabidopsis plants and wild type. Structural identifications of the compounds were essentially based on MS/MS fragmentation data. The structural elucidation of compounds 1–3 is reported in SM, whereas structural characterization of compounds 4–21 has previously been reported (Tsuji *et al*., 2015).

Phenolic metabolite profiling of inflorescence stems from seven individual *amyDFG10* plants and from ten individual *amyDFG12*,* ppiDFG1* and wild‐type plants was carried out using UHPLC‐MS analysis. As many as 1286 different mass signals were detected in all replicates of at least one genotype. ANOVA tests showed that 33 of these signals were present at significantly different intensities between the lines (*P *< 0.05). *Post hoc* analysis showed that all significant differences could be assigned to differences between the *amyDFG10* line and the wild type. No significant differences were observed in signal intensity between the other *LigDFG* lines and wild type. Further analyses demonstrated that the 33 mass signals were derived from a total of 26 different compounds (Table 1, Figure 6). The abundance of 24 of these compounds was higher, and the abundance of 2 compounds was lower in the *amyDFG10* line than in the wild‐type plants (Table 1). Twenty‐one of the 24 compounds that accumulated in the *amyDFG10* line were structurally characterized based on their MS/MS fragmentation patterns. The detailed structural characterization of compounds **1** to **3** is described in the Supporting Information (Figure S3) whereas structural characterization of compounds **4** to **21** was based on literature data (Tsuji *et al*., 2015).

**Table 1:** Phenolic compounds which show significant differences between their abundance in *LigDFG* transgenic Arabidopsis plants and wild type

nr	*m*/*z*	RT (min)	Identity	WT	*amyDFG10*	*amyDFG12*	*ppiDFG1*	Ratio of *amyDFG10*/WT
Compounds with increased abundance in amyDFG10								
HPV‐like compounds								
1	357.12	4.50	HPV + hexose	1569	8751	1502	2062	5.58
2	387.13	5.01	HPS + hexose	240	1944	187	196	8.10
3	399.16	6.45	HPV + hexose + acetate	2568	6277	2365	2000	2.44
Dilignols and dilignol hexosides								
4	373.13	11.85	Gox(β‐O‐4)G	1838	10 382	2079	2127	5.65
5	371.11	14.26	Gox(β‐O‐4)G′	246	2020	132	405	8.21
6	581.19	8.42	Gox(β‐O‐4)G 4‐O‐hexoside (formic acid adduct)	28	2616	32	19	93.43
Neolignan‐like compounds								
7	549.16	8.68	Gox(β‐O‐4)FA + hexose	1126	8516	562	982	7.56
8	549.16	9.19	Gox(β‐O‐4)FA + hexose	5	1634	4	4	326.80
9	549.15	9.73	Gox(β‐O‐4)FA + hexose	470	4759	313	375	10.13
10	503.12	12.21	Gox(β‐O‐4)FA + malate	1218	11 972	1039	1351	9.83
11	503.12	12.85	Gox(β‐O‐4)FA + malate	639	6681	559	636	10.46
12	665.17	9.06	Gox(β‐O‐4)FA + malate + hexose	6	1358	7	0	226.33
13	665.17	9.21	Gox(β‐O‐4)FA + malate + hexose	354	6307	185	384	17.82
14	773.23	14.02	Gox(β‐O‐4)FA + hexose + sinapic acid	1897	21 354	2273	2520	11.26
15	773.23	15.36	Gox(β‐O‐4)FA + hexose + sinapic acid	43	5128	123	79	119.26
16	935.29	11.14	Gox(β‐O‐4)FA + hexose + hexose + sinapic acid	94	5794	114	244	61.64
17	935.29	11.24	Gox(β‐O‐4)FA + hexose + hexose + SA	63	4159	11	173	66.02
18	935.29	11.71	Gox(β‐O‐4)FA + hexose + hexose + SA	17	1982	0	75	116.59
19	516.15	10.13	Gox(β‐O‐4)FA + glutamic acid	17	1459	40	18	85.82
20	417.12	12.29	Gox(β‐O‐4)SA + malate (‐malate)	419	1620	320	382	3.87
21	417.12	13.22	Gox(β‐O‐4)SA + malate (‐malate)	218	1367	193	181	6.27
Compounds with unknown identity								
22	225.04	3.47	Unknown	393	1010	333	417	2.57
23	385.12	12.17	Unknown	49 607	86 594	43 207	55 871	1.75
24	387.15	8.74	Unknown	2275	6362	2198	2226	2.80
Compounds with decreased abundance in amyDFG10								
Compounds with unknown identity								
25	419.14	8.57	Unknown	1583	394	1319	1264	0.25
26	389.13	8.88	Unknown	4931	1970	3762	3823	0.40

All significant differences in abundance were observed between the *amyDFG10* line and wild type. The ratio represents the fold‐change in abundance between the listed transgenic line as compared to wild type. s.d., standard deviation; HPV, hydroxypropiovanillone; HPS, hydroxypropiosyringone; G, guaiacyl unit; Gox, α‐keto‐oxidized guaiacyl unit. The chemical structures of compounds 1–21 are shown in Figure 6.

Several phenolic compounds with an α‐keto functionality were found in the *amyDFG10* plants. The majority of these compounds were dimers of an α‐keto‐G (Gox) unit β–O–4‐linked to either a G unit (**4** and **6**), a G′ unit (derived from coniferaldehyde; **5**), a ferulic acid moiety (**7**–**19**) or to a sinapic acid moiety (**20** and **21**). The dimers were found to be further conjugated with hexose, malic acid, an unknown constituent of 224 Da, glutamate or a combination thereof. The oxidized dimers were also found in plants expressing only *LigD* (Tsuji *et al*., 2015), and their formation was therefore assigned as resulting from LigD activity. The putative LigD substrates giving rise to formation of the oxidized dimers detected in this study were previously identified in Arabidopsis inflorescence stems (Matsuda *et al*., 2010; Morreel *et al*., 2014; Vanholme *et al*., 2012). G(β‐O‐4)ferulic acid and G(β‐O‐4)sinapic acid, and their conjugates are classified as neolignan‐like compounds (Davin and Lewis, 2005) as they share the same structures seen in neolignans, but they are likely produced in the cytosol (Dima *et al*., 2015).

GS‐HPV is the product formed by LigF, but it was not detected in any of the extracts from the transgenic lines. HPV is the final product of the LigDFG pathway and was indeed present as a γ‐*O*‐glucoside conjugate (**1**) and as a γ‐*O*‐acetyl hexoside conjugate (**3**) in extracts of the *amyDFG10* line. In addition, hydroxypropiosyringone (HPS) hexose (**2**) was detected in the *amyDFG10* line, hinting that LigDFG can also act on (β‐O‐4)‐linked S units. Notably, the HPV γ‐*O*‐hexoside (**1**) was also increased in plants expressing only LigD without an apoplastic target signal peptide. This compound was marked as ‘Unknown C_16_H_21_O_9_’ in a previous study involving expression of *LigD* in Arabidopsis (Tsuji *et al*., 2015). Re‐evaluation of the data presented in the latter study showed that the amount of HPS γ‐*O*‐hexoside (**2**) in plants expressing only *LigD* also had a tendency of being increased whereas the level of HPV γ‐*O*‐acetyl hexoside was not significantly increased (Figure S4). In any case, the reported fold‐change as compared to wild type of these HPV‐like compounds was lower in the *LigD*‐expressing lines compared to the *amyDFG10* line (Figure S4, Table 1). It is important to note that the α‐keto‐oxidized G(β‐O‐4)G units were detectable in wild‐type plants as well, suggesting that wild‐type plants do have LigD activity. Furthermore, hexosylated HPV was likewise detected in wild‐type stems, indicating that wild‐type Arabidopsis also exhibits LigF and LigG activity.

### Glucose release

**Figure 7:**
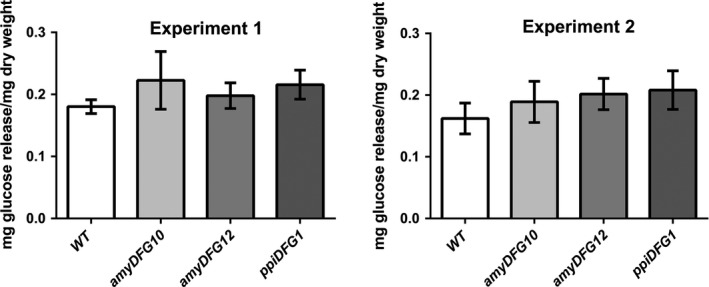
Analysis of saccharification yield in *LigDFG* transgenic Arabidopsis plants. Two‐way ANOVA showed statistically significant increases in glucose release from *amyDFG10* (*P* < 0.02), *amyDFG12* (*P* < 0.02) and *ppiDFG1* (*P* < 0.001), with no interaction between genotype and growth cohort (two cohorts, *n* = 8 in each cohort).

The *LigDFG* transgenic Arabidopsis plants were tested for cell wall saccharification yield. To measure the effects of rather subtle cell wall compositional differences between transgenic and wild‐type lines, partial glucose release was accomplished using a mild enzyme based saccharification procedure. Two growth cohorts of each eight biological replicates of ground dried stems from each of the transgenic Arabidopsis lines (*amyDFG10*,* amyDFG12* and *ppiDFG1*) and from wild type were analysed (Figure 7). Two‐way ANOVA showed statistically significant increases in glucose release from *amyDFG10* (*P* < 0.02), *amyDFG12* (*P* < 0.02) and *ppiDFG1* (*P* < 0.001), with no interaction between genotype and growth cohort.

## Discussion

**Figure 8:**
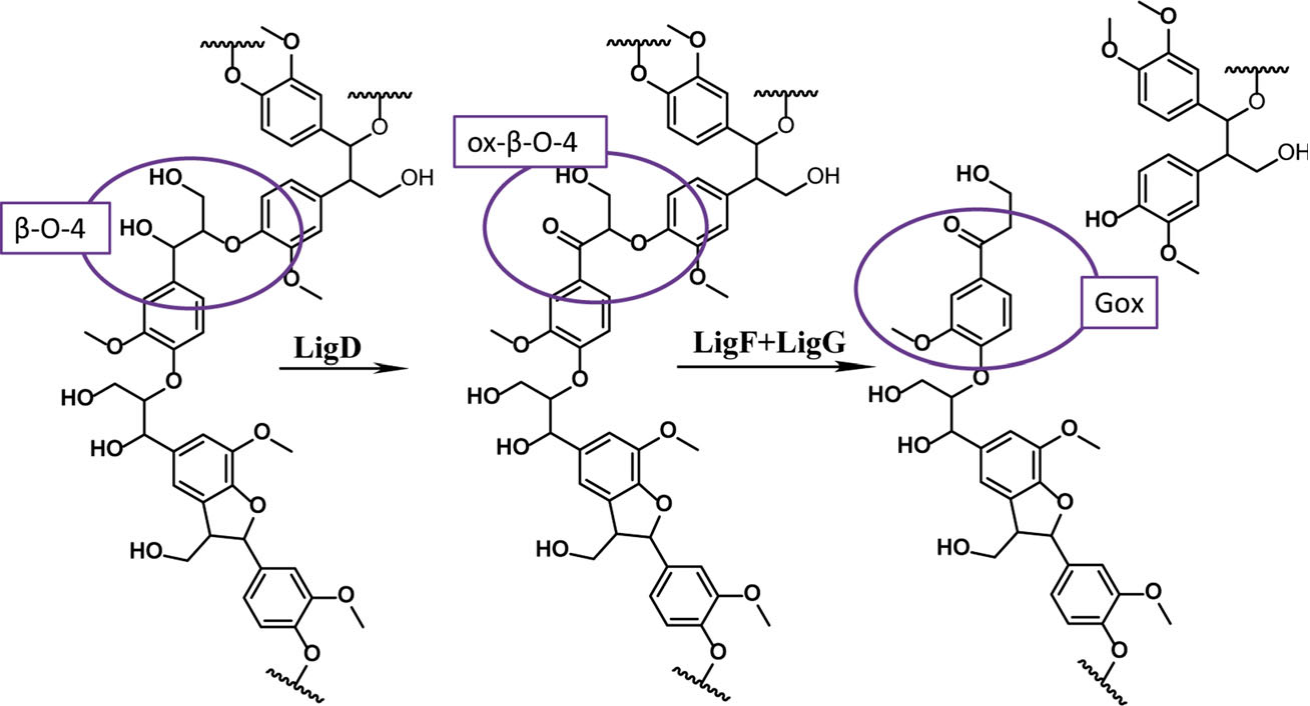
Cleavage of a β–O–4‐bond in lignin by the LigDFG enzymes depicting their theoretical contribution to the observed increase in Gox units as revealed by the 2D HSQC NMR spectra.

Lignin embedded in the plant cell wall impedes enzymatic lignocellulose deconstruction for biofuel production. In this study, we targeted lignin‐degrading Lig enzymes from the bacterium *S. paucimobilis* to the apoplast of Arabidopsis with the aim of modifying lignin structure in a manner that would improve the accessibility of polysaccharide‐hydrolysing enzymes without influencing the overall content of lignin. The overall lignin composition, in terms of G and S units and linkage types, was not changed in the transgenic *LigDFG* Arabidopsis lines, except that the degree of α‐oxidation of G and S units was approximately twofold increased, as deduced from the aromatic regions of the 2D HSQC NMR spectra (Figures 4 and S3). The detected changes in lignin structure were significant and displayed little variation between the individual transgenic plant lines obtained. In Arabidopsis plants expressing only *LigD*, an increase in oxidized β–O–4‐ether structures (Aox structures in Figure 5) was observed consistent with the action of LigD on the Cα‐OH of β–O–4‐linked units (A structures in Figure 5) as exemplified in Figure 8 (Tsuji *et al*., 2015). Upon expression of the entire LigDFG pathway, the LigD product serves as substrate for LigF, and therefore, the oxidized β–O–4‐ether structures (Aox) are further metabolized. In the *LigDFG* lines, the observed increase in the content of oxidized structures (Gox and Sox units) in the absence of elevated levels of oxidized β–O–4‐ether structures (Aox) (Figure 4) demonstrated that the entire LigDFG pathway was functional: the set of LigDFG enzymes reductively cleaved some of the β–O–4‐units resulting in an increase in HPV‐like end‐units. Naturally occurring benzaldehyde and benzoic acid end‐units will contribute to the intensity of Gox and Sox signals (Boerjan *et al*., 2003; Rahimi *et al*., 2013). However, LigDFG activity would not influence the content of, nor modify, these groups and therefore the increase in Gox and Sox in the transgenic *LigDFG* lines was derived from reductive cleavage of α‐oxidized β–O–4‐units. The unaltered Aox/A ratio demonstrated that under the given experimental conditions, the LigD‐step was rate limiting, that is those G units with β–O–4‐bonds that are oxidized to α‐ketones by LigD are cleaved by the action of LigF and LigG.

The observed reductive cleavage of some of the β–O–4‐linked lignin units in the transgenic *LigDFG* lines caused a moderate, but significant, increase in the digestibility of the cell wall by polysaccharide‐hydrolysing enzymes. In a recently published study, Tsuji *et al*. (2015) did not detect increased saccharification yields in transgenic Arabidopsis plants expressing *LigD* alone, although it was expected that the increase in oxidized β–O–4‐structures should render the lignin more easily degradable by alkaline pretreatment. Similarly to the present study, only minor changes in lignin structure were observed by 2D HSQC NMR analysis, although this might reflect issues with respect to achieving apoplastic localization of the enzyme. At the current stage, it may thus be concluded that expression of the entire LigDFG pathway with the amy and ppi signal peptides may be more efficient in terms of achieving increased enzymatic cell wall decomposition. The data seem to suggest that the cleavage of the oxidized β–O–4‐structures (Aox) achieved by expressing LigDFG is needed to improve the saccharification, not merely the oxidation of the β–O–4‐structures.

Phenolic profiling of the transgenic *amyDFG10* Arabidopsis line demonstrated accumulation of dimers oxidized at the Cα position. This is consistent with LigD activity. In agreement with previously reported observations (Tsuji *et al*., 2015), the detected compounds were mainly oxidized G units linked with other G units or with ferulic acid and their hexose and malate derivatives. Such phenylpropanoid dimers are classified as neolignans or lignan‐like and are thought to be involved in plant defence mechanisms rather than in the lignification process (Davin and Lewis, 2005). The cellular localization of this coupling reaction is not known. In lignin formation, oxidative coupling of monolignols takes place in the cell wall by radical reactions catalysed by the action of oxidases (peroxidases or laccases) (Boerjan *et al*., 2003). The G(β‐O‐4)ferulic acid is unlikely to be formed in the cell wall as it would mean that such units would subsequently be incorporated into lignin, but no or very little cell wall‐bound ferulic acid can be detected in dicots (Vogel, 2008). It has recently been demonstrated that radical coupling may also proceed in the cytosol (Dima *et al*., 2015; Niculaes *et al*., 2014) and peroxidases are also found in the vacuole (Welinder *et al*., 2002; Weng and Chapple, 2010). However, as glycosylation is a cytoplasmic process and the vacuole is a storage compartment for glycosylated monolignols (Dima *et al*., 2015; Liu *et al*., 2011), the radical coupling of monolignols to form neolignan‐like compounds most likely takes place in the cytosol before glycosylation and transport into the vacuole. This is corroborated by the observation that the phenolic alterations observed by Tsuji and co‐workers in both purely cytoplasmic and mixed cytoplasmic/apoplastic localized LigD‐expressing transformants are also neolignan‐like compounds (Tsuji *et al*., 2015). Malate (trans‐) esterification occurs inside the vacuole (Hause *et al*., 2002; Strack and Sharma, 1985).

In this study, the LigDFG proteins were targeted to the secretory pathway using well‐proven signal peptides (Liu *et al*., 2004; Rogers, 1985), although leakage or mis‐targeting cannot be fully excluded. The presence of oxidized neolignan‐like compounds such as Gox(β‐O‐4)FA in the transgenic *amyDFG10* Arabidopsis line indicates that the LigD enzyme and G(β‐O‐4)ferulic acid have at some point been present in the same cellular compartment. This could either be caused by mis‐targeting of LigD to the cytoplasm in the *amyDFG10* line, or by co‐occurrence of the LigD enzyme and its substrate G(β‐O‐4)ferulic acid as the protein travels through the secretory pathway to the apoplast. The former explanation is the most likely due to similarities in phenolic profiling with the cytoplasmically localized LigD (Tsuji *et al*., 2015). Quantification of the LigD enzyme in soluble protein extracts from all three *LigDFG*‐expressing lines using Western blot analysis (Figure 3) demonstrated that the expression level of *LigD* was strongly variable between replicate plants from the two homozygous transgenic lines (*amyDFG12* and *ppiDFG1*), but high and more stable in the transgenic *amyDFG10* line, potentially due to the mixed localization of LigD in *amyDFG10* lines. All three transgenic Arabidopsis lines possessed LigDFG activity in the *in vitro* assay, expressed the LigD enzyme to a level detectable by Western blot and displayed benzylic oxidation in β–O–4‐structures. However, two of the lines (*amyDFG12* and *ppiDFG1*) did not display a significant change in soluble phenolics, supporting the notion that the absolute levels of intracellular enzyme accumulation in these lines were negligible. Furthermore, the required cofactors [nicotinamide adenine dinucleotide (NAD^+^) and glutathione (GSH)] are expected to be nonlimiting in the cytosol, meaning that if LigDFG had been present in this compartment in the *amyDFG12* and *ppiDFG1* lines, we would have detected changes in soluble phenolics. The fact that we did not strongly implies that the detected oxidation of G and S lignin units (Gox and Sox in the NMR) and the proposed cleavage of β–O–4‐bonds had indeed taken place in the apoplast.

Two cofactors are required for the ability of LigDFG enzymes from *S. paucimobilis* to catalyse cleavage of the β‐aryl ether units in lignin. LigD activity is dependent on the presence of NAD^+^ whereas LigF and LigG activity requires the presence of GSH. The concentration of these two cofactors is expected to be low in the apoplast although NAD^+^ is detected in the apoplastic fluid and associated with peroxidase activity (Otter and Polle, 1997) and GSH is involved in detoxification processes and small amounts are found in the apoplast (Foyer *et al*., 2001). Low concentrations of cofactors are therefore considered to be the limiting factor for LigDFG activity in the apoplast rather than the level of the LigDFG proteins. A completely separate factor that may also restrict the level of modifications introduced into lignin by the expression of *LigDFG* is that LigD and LigF show high substrate specificity with only one of four stereoisomers of G(β‐O‐4)G being accepted as substrate and cleaved.

The results obtained from the characterization of the transgenic Arabidopsis plants expressing *LigDFG* provided additional knowledge on the substrate specificity of LigDFG. The 2D HSQC NMR analysis showed an increase in the amount of Sox units formed. In addition, S(β‐O‐4)G was converted into HPS by the LigDFG enzymes. The phenolic analysis also revealed formation of Gox(β‐O‐4)sinapic acid. This demonstrated that coniferyl alcohol dimers as well as sinapyl alcohol and sinapic acid ether dimers were recognized by the LigDFG enzymes. These *in vivo* activities match recently reported *in vitro* activities of LigD on S(β‐O‐4) units (Gall *et al*., 2014b; Tsuji *et al*., 2015). The ability of the LigDFG enzymes to reductively cleavage not only G units expands the potential of using this approach to modify lignin structure in plants as this allows a greater variety of ether‐linked units to be cleaved.

Expression of the individual *LigD*,* LigF* and *LigG* genes in transgenic Arabidopsis lines provided interesting insights into the ability of endogenous plant enzyme activities to metabolize intermediates in the LigD‐LigF‐LigG pathway. When protein extracts from *LigF*‐expressing lines were incubated with MPHPV as substrate, HPV was formed in addition to the expected product GS‐HPV (Figure S2). In the bacterial pathway, the conversion of GS‐HPV to HPV is catalysed by LigG. We did not detect LigG activity in enzyme extracts from WT Arabidopsis leaves, even upon prolonged incubation, and based on this it can be suggested that the enzyme responsible for the conversion in the transgenic lines was LigF. However, the phenolic profiling demonstrated that HPV derivatives are indeed present in stems from WT plants, and re‐examination of the results from Tsuji *et al*. (2015) demonstrated that the amounts of these compounds were also slightly increased in plants expressing only LigD. These results suggest that Arabidopsis does harbour enzymes with LigF and LigG activities. The discrepancy in enzyme activity between leaves and stems can be explained either by differential expression of endogenous LigF and LigG‐like enzymes in different tissues or at different growth stages, or by the endogenous activities being collated into one enzyme with LigFG activity. LigG belongs to the omega class of GSTs (Meux *et al*., 2012) that are characterized by catalysing redox reactions and reductive deglutathionylations instead of a classical glutathionylation reactions (Board *et al*., 2008). A cysteine residue in the active site of the omega class of GSTs is essential for their different mode of action. In plants, GSTs of the lambda class are known to have cysteine in the active site as opposed to glutathionylating plant GSTs that harbour a serine in this position (Lallement *et al*., 2014b). The lambda class GSTs from wheat and poplar are able to reduce glutathionylquercetin to quercetin (Dixon and Edwards, 2010; Lallement *et al*., 2014a). A lambda class GST is therefore a good candidate for the LigG activity found in wild‐type Arabidopsis, but no plant enzymes are known which catalyse a LigF‐like reaction. In bacterial degradation of pentachlorophenols, a single enzyme is responsible for a presumably two‐step reaction similar to the combined LigFG‐reaction (Huang *et al*., 2008). The reaction consumes two GSH‐molecules and, if the enzyme is damaged by oxidation at the catalytic Cys‐residue, it produces a GSH‐conjugate that it is unable to cleave. It is therefore possible that under certain conditions, LigF harbours additional LigG activity, and that Arabidopsis produces an enzyme with both activities. Further analysis is required in order to identify the Arabidopsis enzymes and compare their activity to LigF and LigG to elucidate whether the introduction of these enzymes into the apoplast in concert with LigD is sufficient to introduce changes into lignin structure. As endogenous lambda GSTs in plants are targeted to either the cytosol, chloroplast or peroxisome (Dixon *et al*., 2009), such genetic engineering will still be required to obtain degradation of lignin.

In conclusion, we have shown that expression of *LigDFG* encoding the β‐aryl ether‐unit‐degrading enzymes from *S. paucimobilis,* has an impact on lignin structure, thus providing a platform for saccharification yield improvement, a desired feature of plants for biofuel production. Even though there are limitations to the amount of change possible from this approach, we have shown that the changes observed are statistically significant and that it is possible to modify lignin structure, without significant alterations to neolignan‐like compounds, by targeting bacteria‐derived lignin‐degrading enzymes to the secretory pathway. Fine‐tuning of lignin cross‐linking may be combined with other modest modifications of lignin structure offering improved properties with respect to biomass conversion into biofuels without compromising plant robustness.

## Materials and methods

### Vector construction

For expression of the various Lig genes in *E. coli* and *Arabidopsis thaliana*, genes were synthesized by GenScript (Hong Kong). For expression in *E. coli,* the following genes were synthesized: *LigD* (DDBJ accession no. D11473–1), *LigF* (DDBJ accession no. D11473‐2) and *LigG* (DDBJ accession no. AB026292) and subcloned into the pETSTREP vector (Dixon *et al*., 2009). A list of the synthetic gene sequences and the primers used for amplification can be found in Tables S1 and S2.

Vectors for plant expression studies were constructed using the USER cloning technique (Geu‐Flores *et al*., 2007). The gene sequences were codon‐optimized for plant expression and synthesized with added signal peptides, either from barley α‐amylase (amy) (Genbank accession no. K02637) (Rogers, 1985) or from potato proteinase inhibitor II (ppi) (Liu *et al*., 2004). Genes were amplified independently by PCR (Samalova *et al*., 2006) linking the *LigD*,* LigF*,* LigG* sequences with 2A sequences. Each gene was provided with a signal peptide (amy or ppi). The genes were inserted into the PacI/Nt.BbvcI USER cloning site in pCAMBIA1300 USER compatible vectors and controlled by an enhanced 35S promotor (Geu‐Flores *et al*., 2009). Vectors harbouring a single gene (*amy‐LigD*,* amy‐LigF*,* amy‐LigG*,* ppi‐LigD*,* ppi‐LigF*,* ppi‐LigG*) were constructed by ligation of the PCR products into the PacI/Nt.BbvcI USER cassette in p1300 (for *amy‐LigF*,* amy‐LigG*,* ppi‐LigF* and *ppi‐LigG*) or p2300 (*amy‐LigD* and *ppi‐LigD*).

### Plant cultivation

*Arabidopsis thaliana* Col‐0 plants used for protein assays and screening experiments and *N. benthamiana* plants used for transient expression were grown at a long‐day regime (16‐h light) at a temperature of 21 °C and a relative humidity of 60%. For 2D‐NMR, metabolite profiling and saccharification experiments, Arabidopsis plants were grown at a short‐day regime (8 h light) for 10 weeks followed by growth in the long‐day regime. For metabolite profiling, the stems were harvested when they reached a height of approximately 40 cm. For NMR and saccharification experiments, the plants were grown until senescence.

### Transient and stable plant transformation

For plant transformation, expression vectors were introduced into *Agrobacterium tumefaciens* strain Agl1 (Lazo *et al*., 1991) by electroporation (Shen and Forde, 1989). Arabidopsis transformation was performed using the floral dip method (Bent, 2006). Transient expression in tobacco was accomplished by agro‐infiltration (Goodin *et al*., 2002) using 4‐week‐old plants.

### Selection of transformed Arabidopsis plants

Mature seeds were collected from fully senesced Arabidopsis plants. Sterilized seeds were plated on ½ Murashige and Skoog (pH 5.8) containing 30 μg/mL geneticin (G418) or 30 μg/mL hygromycin B. After cold‐stratification (4 °C, 72 h), seeds on plates were incubated at 21 °C, long‐day regime until the transformed seedlings had developed true leaves. The seedlings were transferred to soil and grown in a greenhouse. Transgenic plants were verified by PCR on gDNA isolated from their leaves. Primers used to amplify inserted *LigDFG* genes were LigDfw (5′‐CGCTCACGGCATCGTTCTTGATA‐3′) and LigGrev (5′‐CCACCTTGTGTATAGTCATAG‐3′) (Tm 54 °C, 30 cycles).

The transgenic plants were characterized by NMR, saccharification and phenolic profiling using plants grown from seeds of T3 plants of homozygous *amyDFG12* and *ppiDFG1* lines sown alongside segregating *amyDFG10* seeded plants and wild type. In the phenolic profiling experiment, segregating *amyDFG10* plants were genotyped by PCR for the presence of the amyDFG10 construct. For the experiments with plants expressing only *amyD, amyF, amyG*,* ppiD* and *ppiF,* T3 homozygous lines were used.

### Expression in *E. coli*

LigD, LigF and LigG were expressed in the *E. coli* BL21DE3 strain (New England Biolabs, Ipswich, MA, USA). Bacteria were grown (28 °C) in LB medium (50 mL) containing 50 μg/mL of kanamycin. Gene expression was induced by addition of Isopropyl β‐d‐1‐thiogalactopyranoside (IPTG) (final concentration 1 mm) at OD_600 nm_ 0.6. After the induction (5 h), cells were harvested by centrifugation and protein was isolated.

### Extraction and metabolite profiling of phenylpropanoids

The lower part (8 cm) of Arabidopsis flowering stems (total height of around 40 cm) carrying multiple fully developed siliques was frozen and ground in liquid nitrogen. Sample preparation procedure and UHPLC‐MS setting were previously reported (Vanholme *et al*., 2013b). From the resulting chromatograms, 1286 de‐isotoped constituent signals were integrated and aligned via Progenesis QI (Waters Corporation, Milford, MA), with each constituent being defined by its *m*/*z* value and retention time. Statistics (ANOVA with *post hoc t*‐test) were performed in Progenesis QI extension EZinfo. The following stringent filters were used: abundance > 1000, *P*‐value ANOVA < 0.05. Statistical analyses were performed on arcsinh‐transformed ion intensities. For structural elucidation, MS/MS was used. For MS/MS, all settings were the same as in full MS, except the collision energy was ramped from 15 to 35 eV in the trap, and the scan time was set at 0.5 s.

### Protein isolation

Plant soluble proteins were extracted from rosette leaves of T3 plants by homogenization in liquid nitrogen and extraction into 100 mm potassium phosphate buffer (pH 7.5) including 3 mm EDTA, 1 mm phenylmethyl‐sulfonyl fluoride (PMSF), and 1 mm GSH in the presence of polyvinylpoly‐pyrrolidone (PVPP) (100 mg/1 g of plant material). The homogenate was filtered through muslin gauze and centrifuged (30 min; 20 000 ***g***), and the supernatant obtained used for Western blotting, protein assays, etc.

### LigDFG enzyme assays

Appropriate substrates, GGE (0.5 mm final concentration) or MPHPV (0.5 mm final concentration), NAD^+^ (1 mm final concentration) and GSH (1 mm final concentration) were mixed in 100 mm phosphate buffer (pH 7.5) (total volume: 100 μL), and proteins of bacterial or plant origin were added. Following incubation (16 h, room temperature), the enzyme reaction was stopped by adding MeOH (50% final concentration).

### Chemical synthesis

The chemical synthesis of MPHPV S4 and HPV‐glucopyranoside S14 is described in Data S1.

### Immunodetection by Western blot

Antibodies against LigD were produced by Agrisera (Vännäs, Sweden) in rabbits using the LigD amino acid sequence NH_2_‐CDIMDREAYARAADE‐CONH_2_ (residues 63–76) as an antigen. The serum obtained (1 : 500 dilution) 15 weeks after the first immunization was used for Western blots to obtain a semi‐quantitative assessment of the LigD protein levels in transgenic plants.

### LC‐MS analyses

LC‐MS analyses were performed using a Dionex HPLC fitted with a UV detector (UVD340U with microflow cell) and connected to an MSQplus MS detector (Thermoscientific, Waltham, MA, USA). Chromatographic separation was obtained using a Zorbax SB C18 column (2.1 × 50 mm, Phenomex, Værlose, Denmark). The mobile phases were A: 50 μm NaCl, 0.1% HCOOH; B: 50 μm NaCl, 0.1% HCOOH, 80% MeCN. The gradient used was 0% B to 70% B (linear) in 30 min, 70% B to 100% B in 5 min (flow rate: 0.2 mL/min). The column was rinsed with 100% B and re‐equilibrated with A for 6 min (flow rate: 0.3 mL/min). Mass spectrometry was performed in the positive ionization mode ESI (cone voltage: 75 V, needle voltage: 3.5 kV). Samples were analysed in full‐scan mode (*m*/*z* 100–800).

### 2D HSQC NMR

The Arabidopsis samples (1 g) obtained from the 25 cm lower part of 10 flowering stems (total plant height around 40 cm and carrying multiple fully developed siliques) were used for NMR analyses, and acetylated lignin samples were prepared as described in Bonawitz *et al*. (2014), Lu and Ralph (2003), Tsuji *et al*. (2015).

For NMR investigations, the acetylated lignin sample (55 mg) was dissolved in deuterated chloroform (CDCl_3_, 0.5 mL) in a 5 mm NMR tube. The 2D HSQC spectrum was acquired using a Bruker 700 MHz instrument with a cryogenically cooled QCI (^1^H/^31^P/^13^C/^15^N) 5 mm inverse (^1^H coils closest to the sample) probe using a standard HSQC program. Statistics (ANOVA with *post hoc t*‐test) were performed.

### Saccharification yield

Dried ground Arabidopsis stems (30 mg) obtained from lower 8 cm were resuspended in MilliQ water (600 μL, 70 °C, 2 h), centrifuged (12 000 ***g***, 10 min) and the pellet obtained resuspended in sodium acetate buffer (600 μL, 50 mm, pH 4.5) containing Cellic Ctec2 (5 FPU/g of dry matter; Novozymes, Bagsværd, Denmark) and xylanase BioFeed Wheat (Novozymes, 1% of biomass). Samples were incubated (24 h, 50 °C, shaking), centrifuged (16 000 ***g***, 5 min) and aliquots (20 μL) of the supernatant were hydrolysed (1 h, 120 °C) by addition of trifluoroacetic acid (TFA) (150 μL) dissolved in MilliQ water (830 μL). Solvent was removed under vacuum and the residue obtained dissolved in water (1 mL). Glucose concentration was determined by high‐performance anion exchange chromatography with pulsed amperometric detection (HPAEC‐PAD) using a PA20 column (Thermoscientific, Waltham, MA, USA) (Øbro *et al*., 2004).

## Conflict of interests

The authors declare no conflict of interests.

## Supporting information


**Figure S1** Detection of LigDFG catalysed products and intermediates formed by transient expression of LigDFG in tobacco.

**Figure S2** Enzymatic in vitro assays of LigD, LigF and LigG separately expressed in Arabidopsis.

**Figure S3** MS/MS spectra for the structural elucidation of compounds 1–3 detected by phenolic profiling.

**Figure S4** Targeted search for the presence of HPV γ‐O‐hexoside, HPS γ‐O‐hexoside and HPV γ‐O‐acetyl hexoside in LigD‐expressing Arabidopsis plants and re‐evaluation of literature data (Tsuji *et al*., 2015).

**Table S1** Synthetic LigDFG gene sequences used in the study.

**Table S2** List of primers used in the study for vector construction.

**Table S3** 2D HSQC NMR. Lignin aromatic unit and interunit linkage distribution (integrals only). The structures referred to by A to E, Aox, G, Gox, S, Sox are those defined in Figures 4 and 5.

**Data S1** Chemical synthesis of MPHPV and HPV‐glucopyranoside.
