# Supplementary figures and images for: Degradation of lignin β‐aryl ether units in Arabidopsis thaliana expressing LigD, LigF and LigG from Sphingomonas paucimobilis SYK‐6
Source: Plant Biotechnol J. 2016 Nov 29;15(5):581–93. doi: 10.1111/pbi.12655 (PMC5399005; doi:10.1111/pbi.12655)

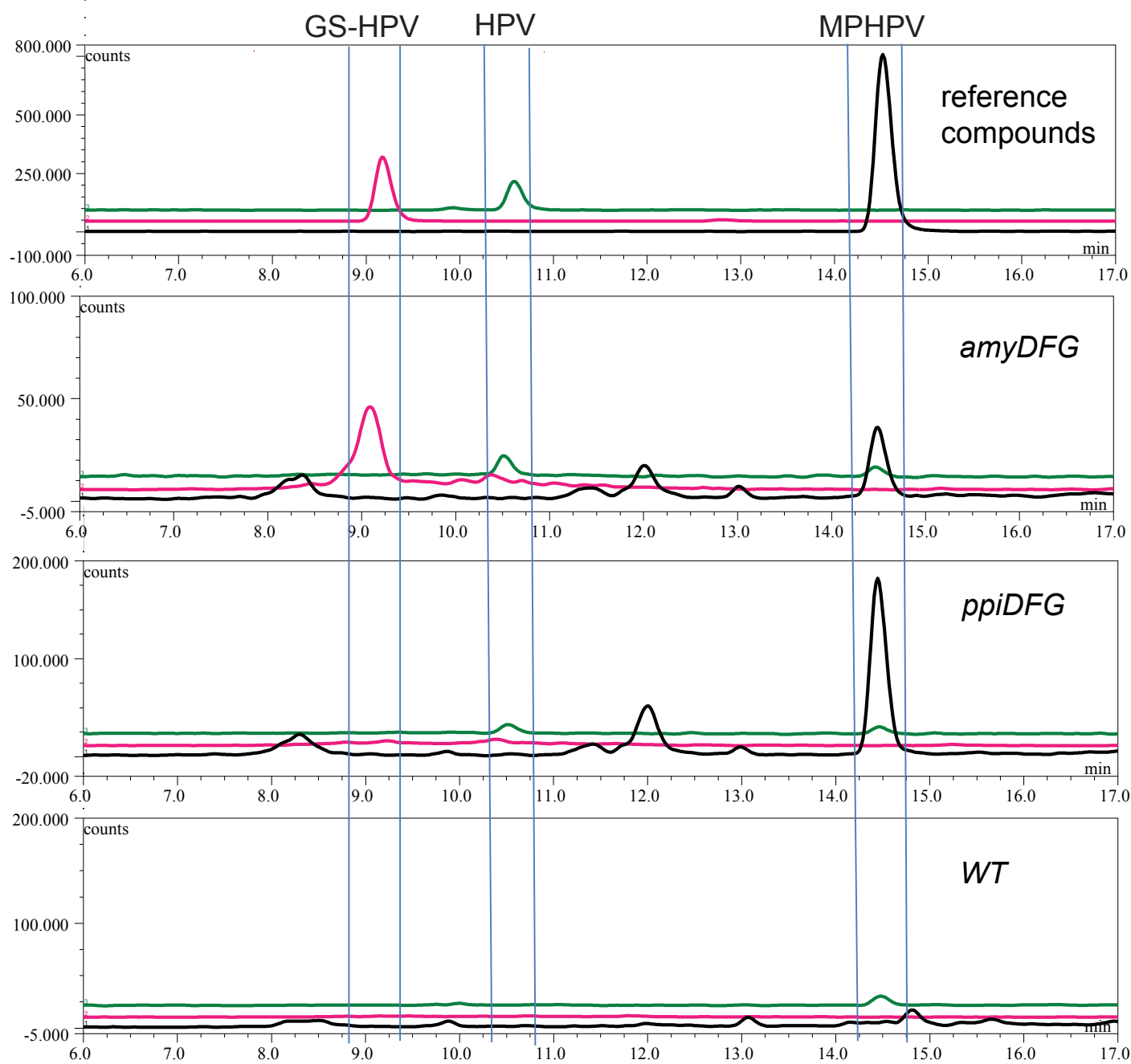

Supplement: Supplementary file 1 — Figure S1 Detection of LigDFG catalysed products and intermediates formed by transient expression of LigDFG in tobacco. [file PBI-15-581-s004.pdf]

GS-HPV

HPV

MPHPV

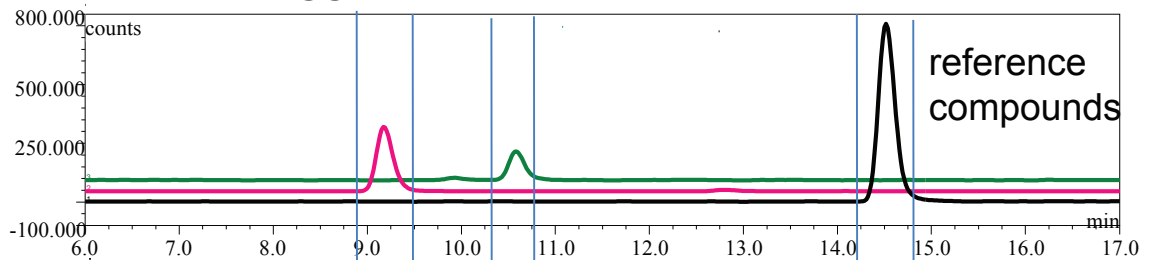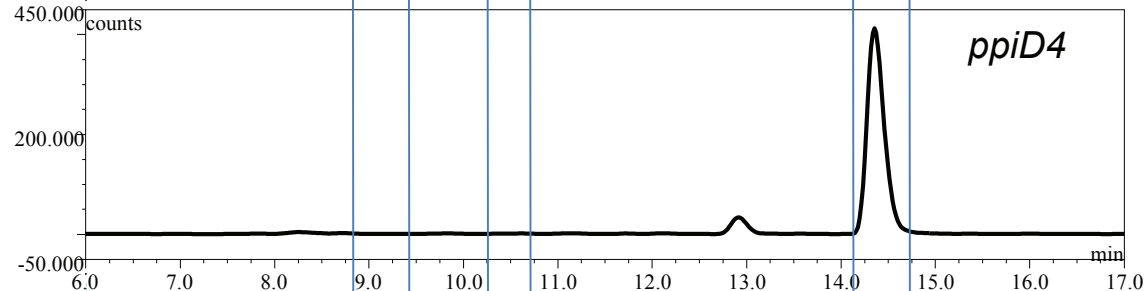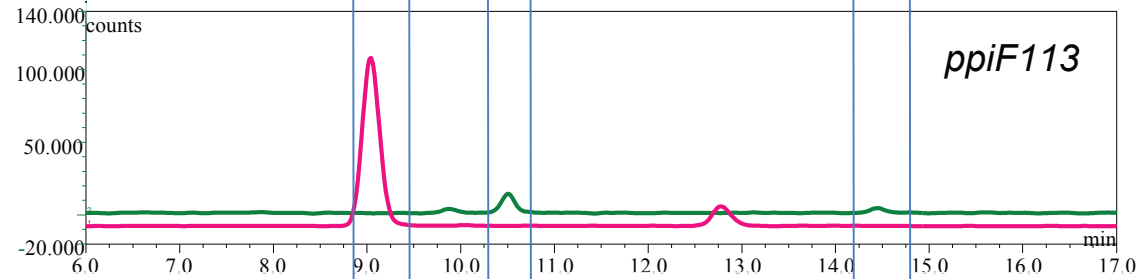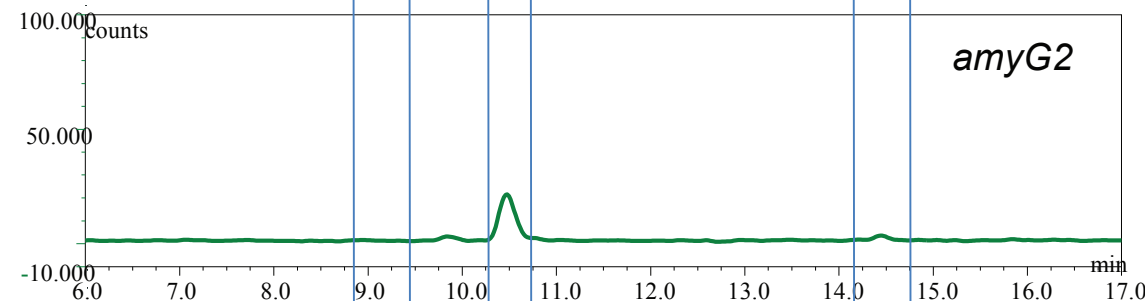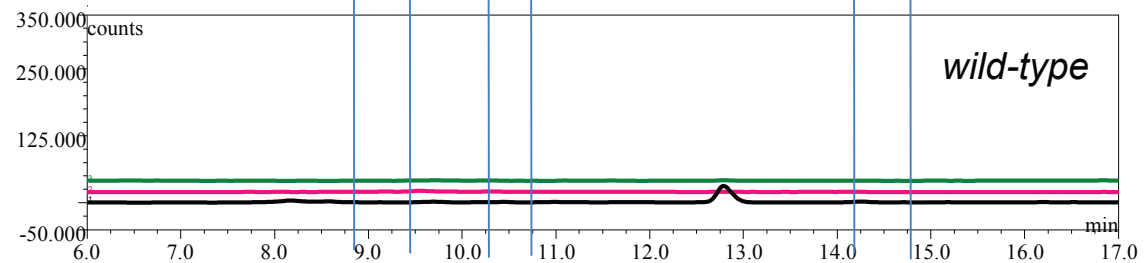

Supplement: Supplementary file 2 — Figure S2 Enzymatic in vitro assays of LigD, LigF and LigG separately expressed in Arabidopsis. [file PBI-15-581-s006.pdf]
