# Supplementary material for: Degradation of lignin β‐aryl ether units in Arabidopsis thaliana expressing LigD, LigF and LigG from Sphingomonas paucimobilis SYK‐6
Source: Plant Biotechnol J. 2016 Nov 29;15(5):581–93. doi: 10.1111/pbi.12655 (PMC5399005; doi:10.1111/pbi.12655)
Supplement: Supplementary file 3 — Figure S3 MS/MS spectra for the structural elucidation of compounds 1–3 detected by phenolic profiling. [file PBI-15-581-s005.pdf]

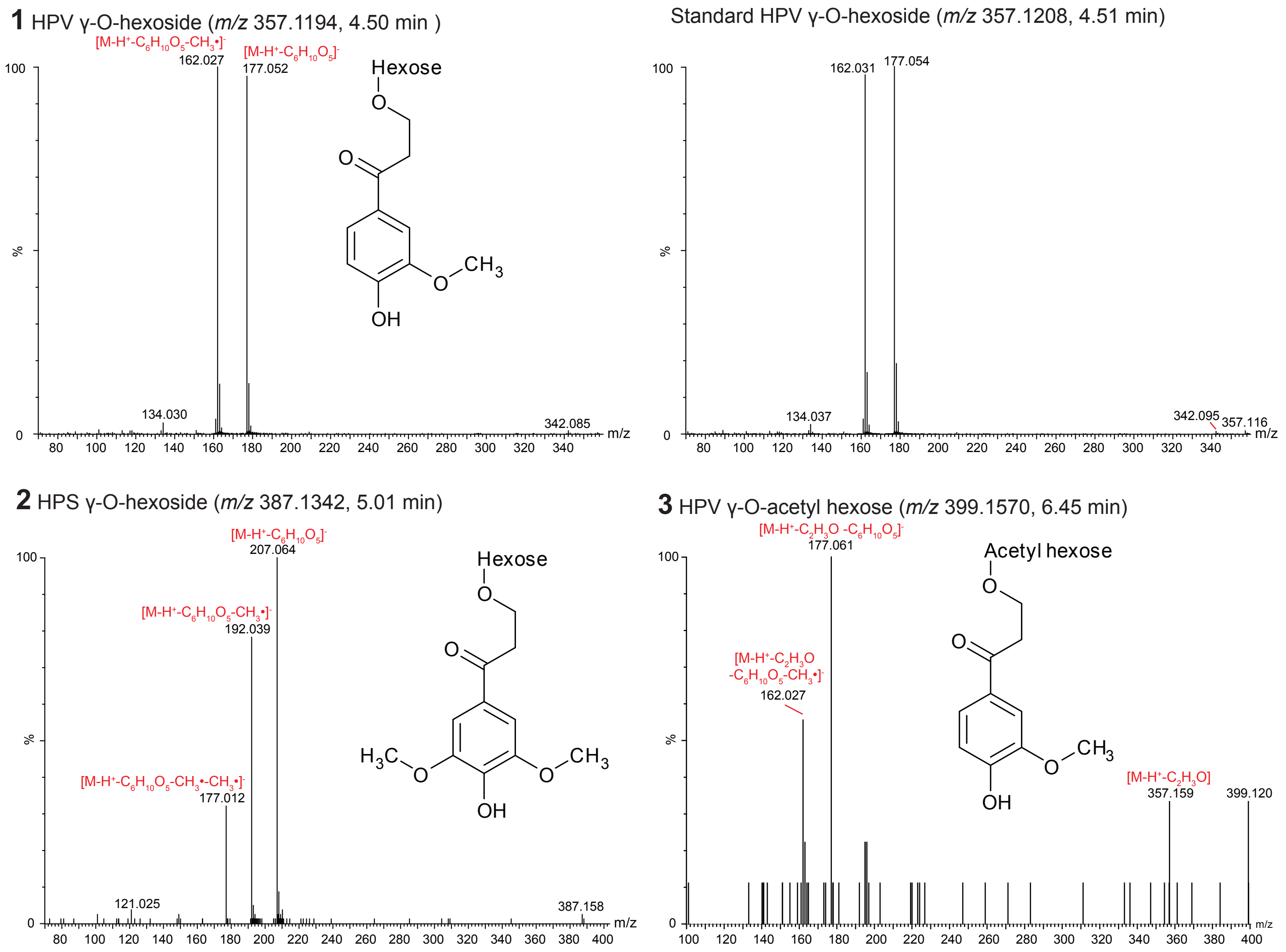

1 HPV γ-O-hexoside (m/z 357.1194, 4.50 min)
[M-H+-C6H10O5-CH3•]-
162.027
[M-H+-C6H10O5]-
177.052
134.030
342.085
Hexose
O
O
O
CH3
OH
100
%
0
m/z
80 100 120 140 160 180 200 220 240 260 280 300 320 340
Standard HPV γ-O-hexoside (m/z 357.1208, 4.51 min)
162.031
177.054
134.037
342.095
357.116
2 HPS γ-O-hexoside (m/z 387.1342, 5.01 min)
[M-H+-C6H10O5]-
207.064
[M-H+-C6H10O5-CH3•]-
192.039
[M-H+-C6H10O5-CH3•-CH3•]-
177.012
121.025
387.158
H3C
CH3
80 100 120 140 160 180 200 220 240 260 280 300 320 340 360 380 400
3 HPV γ-O-acetyl hexose (m/z 399.1570, 6.45 min)
[M-H+-C2H3O -C6H10O5]-
177.061
Acetyl hexose
[M-H+-C2H3O
-C6H10O5-CH3•]-
162.027
[M-H+-C2H3O]
357.159
399.120
100 120 140 160 180 200 220 240 260 280 300 320 340 360 380 400
