# Supplementary material for: Degradation of lignin β‐aryl ether units in Arabidopsis thaliana expressing LigD, LigF and LigG from Sphingomonas paucimobilis SYK‐6
Source: Plant Biotechnol J. 2016 Nov 29;15(5):581–93. doi: 10.1111/pbi.12655 (PMC5399005; doi:10.1111/pbi.12655)
Supplement: Supplementary file 4 — Figure S4 Targeted search for the presence of HPV γ‐O‐hexoside, HPS γ‐O‐hexoside and HPV γ‐O‐acetyl hexoside in LigD‐expressing Arabidopsis plants and re‐evaluation of literature data (Tsuji et al., 2015). [file PBI-15-581-s007.pdf]

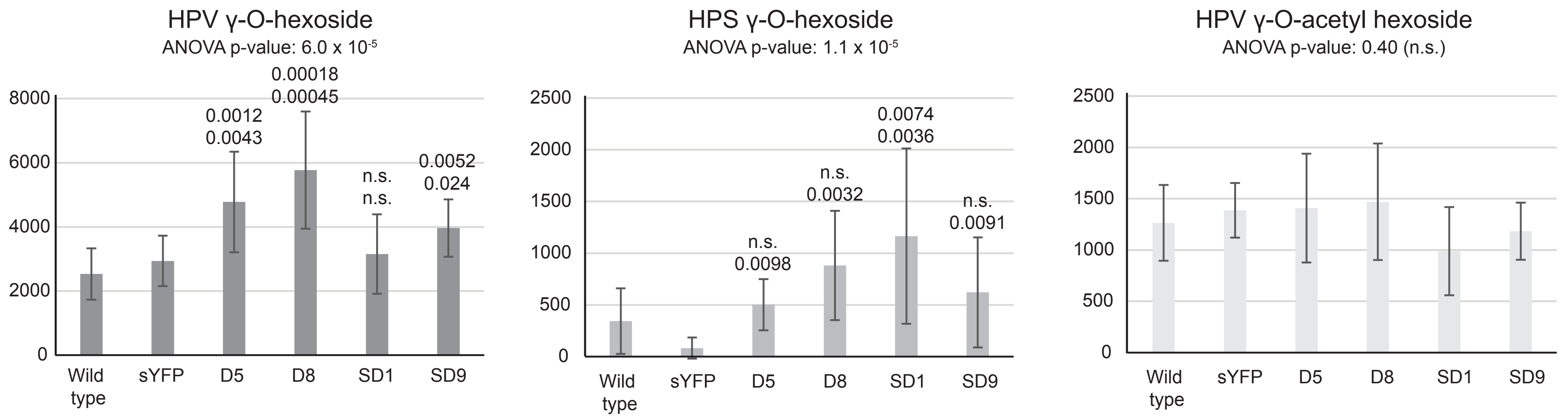
HPV γ-O-hexoside
ANOVA p-value: 6.0 x 10-5
8000
6000
4000
2000
0
0.0012
0.0043
0.00018
0.00045
n.s.
n.s.
0.0052
0.024
Wild type
sYFP
D5
D8
SD1
SD9
HPS γ-O-hexoside
ANOVA p-value: 1.1 x 10-5
2500
2000
1500
1000
500
0
n.s.
0.0098
n.s.
0.0032
0.0074
0.0036
n.s.
0.0091
Wild type
sYFP
D5
D8
SD1
SD9
HPV γ-O-acetyl hexoside
ANOVA p-value: 0.40 (n.s.)
2500
2000
1500
1000
500
0
Wild type
sYFP
D5
D8
SD1
SD9
