# Supplementary material for: Degradation of lignin β‐aryl ether units in Arabidopsis thaliana expressing LigD, LigF and LigG from Sphingomonas paucimobilis SYK‐6
Source: Plant Biotechnol J. 2016 Nov 29;15(5):581–93. doi: 10.1111/pbi.12655 (PMC5399005; doi:10.1111/pbi.12655)
Supplement: Supplementary file 8 — Data S1 Chemical synthesis of MPHPV and HPV‐glucopyranoside. [file PBI-15-581-s008.pdf]

The chemical synthesis of MPHPV S4 [α-(2-methoxyphenoxy)-β-hydroxypropiovanillone (S4)] was accomplished from commercially available GGE S1 [(guaiacylglycerol-β-guaiacyl ether (S1)] as shown in Figure 1A. The phenolic OH- and primary OH-groups of S1 were selectively protected with a tert-butyldiphenylsilyl group via silylation with tert-butyldiphenylchlorosilane using the DMAP-TEA method (Chaudhary and Hernandez, 1979) to give S2 in quantitative yield. Oxidation of the remaining free secondary OH-group of S2 with periodinane (Dess and Martin, 1991) afforded the ketone S3 in 90 % yield. Finally, removal of the tert-butyldiphenylsilyl groups with TBAF (tetrabutylamonium fluoride) in THF (Hanessian and Lavallee, 1975) afforded the desired product S4 in 85 % yield.

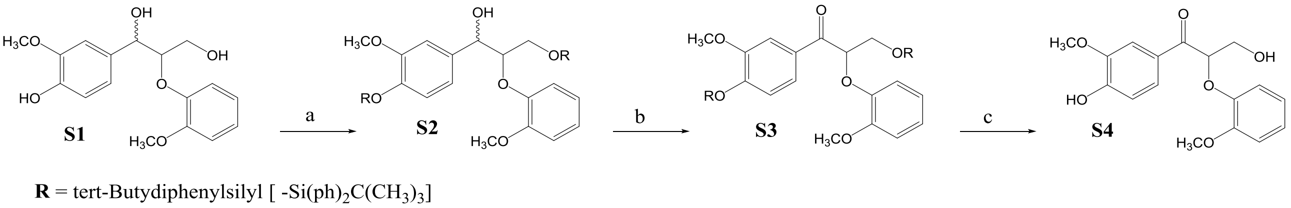

Figure 1. Chemical synthesis of MPHPV (α-(2-methoxyphenoxy)-β-hydroxypropiovanillone) from GGE. The reagents and reaction conditions used were: a) TEA, DMAP, TBDPSi-Cl, CH_2Cl_2, r.t., 1 h, 97 %; b) Dess–Martin periodinane, 90% ; c) TBAHF/THF, r.t. 1 h, 85 %.

The chemical synthesis of HPV-glucopyranoside S14 (3-(β-D-glucopyranosyl)oxy-1-(4-hydroxy-3-methoxyphenyl)-propan-1-one (S14)) is outlined in Figure 2.

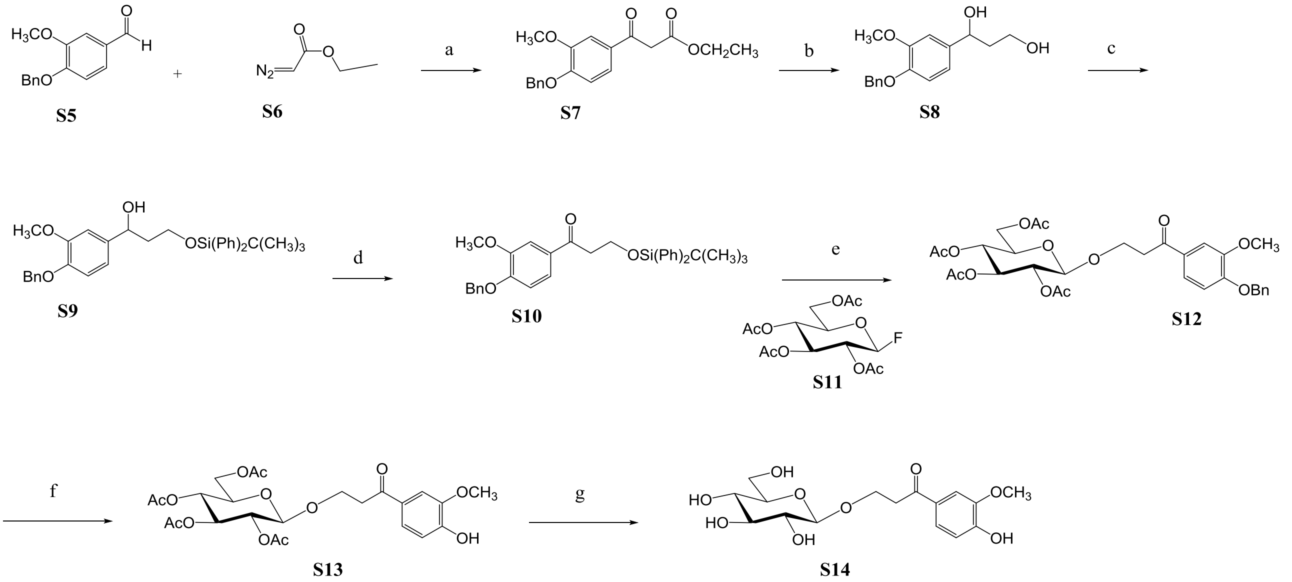

Figure 2. Chemical synthesis of HPV-glucopyranose from 4-benzyloxy-3-methoxybenzaldehyde

Compound nomenclature:

S**5**: 4-Benzyloxy-3-methoxybenzaldehyde; S**6**: Ethyl diazoacetate; S**7**: Ethyl 3-(4-benzyloxy-3-methoxyphenyl)-3-oxopentanoate; S**8**: 3-(4-Benzyloxy-3-methoxyphenyl)-3-hydroxy-propan-1-ol;

S**9**: 1-(4-Benzyloxy-3-methoxyphenyl)-3-(*tert*-butyldiphenylsilyl)oxy-popan-1-ol;

S**10**: 1-(4-Benzyloxy-3-methoxyphenyl)-3-(*tert*-butyldiphenylsilyl)oxy-popan-1-one;

S**11**: 2,3,4,6-Tetra-*O*-acetyl-β-D-glucopyranosyl fluoride;S**12**: 1-(4-Benzyloxy-3-methoxyphenyl)-3-(2,3,4,6-tetra-*O*-acetyl-β-D-glucopyranosyl)oxy-propan-1-one;

S**13**:3-(2,3,4,6-Tetra-*O*-acetyl-β-D-glucopyranosyl)oxy-1-(4-hydroxy-3-methoxyphenyl)-propan-1-one;

S**14**:3-(β-D-glucopyranosyl)oxy-1-(4-hydroxy-3-methoxyphenyl)-propan-1-one(HPV-Glucoside);

The reagents and reaction conditions used were: a) 5 mol % $NbCl_5$, CH_2Cl_2, r.t; b) $NaBH_4$ (4 eq), MeOH, r.t; c) TEA, DMAP, TBDPSi-Cl, CH_2Cl_2, r.t.; d) Dess–Martin periodinane, 90% ; e), $BF_3.Et_2O$, CH_2Cl_2, r.t., 1.5 h; f) $AlCl_3$-$PhNMe_2$, CH_2Cl_2, r.t; g) $NaOCH_3$-MeOH, MeOH, r.t., Dowex (H^+ Form).

In this synthesis, commercially available 4-benzyloxy-3-methoxybenzaldehyde (S**5**) was treated with ethyl diazoacetate (S**6**) in the presence of $NbCl_5$ as described for similar aldehydes (Yadav et al., 2005) to give the corresponding β-ketoester S**7** in 75 % yield. Chemical modification of S**7** via reduction (Chaudhuri et al., 2010), selective silylation (Chaudhary and Hernandez, 1979) and oxidation (Dess and Martin, 1991) afforded compound S**10**. The silylated derivative S**10** was subjected for glycosylation with glycosyl fluoride (S**11**) (Zagrobelny et al., 2014) using borotrifluoroetherate as promoter (Kunz and Sager, 1985) to afford the protected glucoside S**12.** S**12** was debenzylated (Akiyama et al., 1992) to give S**13** from which the acetyl groups linked to the sugar moiety were removed using standard procedures afforded the target glucoside S**14**. The yield obtained in each of the deprotection steps was low and may be optimized. Structural verification of the synthesized compounds was carried out using electrospray ionisation mass spectrometry and ^1H, ^{13}C-NMR experiments.
